# Optimization of Postoperative Antimicrobial Therapy in Surgical Patients Using a Clinical Decision Support System: Use Patterns and Clinical Outcomes

**DOI:** 10.3390/medicina61112043

**Published:** 2025-11-15

**Authors:** Miguel Ángel Amor García, Irene Orozco Cifuentes, Raquel Moreno Díaz, José Antonio Martínez Consuegra, Carmen de Cáceres Velasco

**Affiliations:** 1 Pharmacy Service, Hospital Universitario Infanta Cristina, 9 de Junio, Av. 2, 28981 Parla, Spain; irene.orozco@salud.madrid.org (I.O.C.); rmorenod@salud.madrid.org (R.M.D.); carmen.decaceres@salud.madrid.org (C.d.C.V.); 2 Internal Medicine Service, Hospital Universitario Infanta Cristina, 9 de Junio, Av. 2, 28981 Parla, Spain; jantonio.martinez@salud.madrid.org

**Keywords:** clinical decision support systems, antimicrobial stewardship, surgical patients

## Abstract

*Background and Objectives*: Antimicrobial stewardship plays a key role in the surgical setting by reducing the incidence of healthcare-associated infections and limiting the emergence of antimicrobial resistance. Clinical Decision Support Systems (CDSSs), when integrated into routine practice, are valuable tools for optimizing antimicrobial prescribing. However, evidence regarding their impact on surgical patients, particularly across different specialties, remains limited. *Materials and Methods*: We conducted a quasi-experimental time series study in surgical patients at a primary-level hospital, evaluating the effect of a CDSS on postoperative antimicrobial therapy. The pre-intervention period included patients admitted from April 2017 to September 2020, and the post-intervention period included those admitted from October 2020 to March 2024. Antimicrobial consumption and expenditures were measured as defined daily doses (DDDs) per 1000 patient-days and euros (€) per 1000 patient-days, respectively. Subgroup analyses were performed by the surgical service. Clinical outcomes included mortality and length of stay (LOS). *Results*: Following CDSS implementation, overall antimicrobial consumption decreased by 4.4%. Significant reductions were observed in aminoglycosides (−52.0%), macrolides, lincosamides and streptogramins (−40.6%), and fluoroquinolones (−32.3%). Reductions were heterogeneous across surgical services, with significant reductions in Traumatology (−21.3%) and Urology (−14.3%). Expenditures decreased from 3185.4 to 2733.9€/1000 patient-days (−14.2%; *p* = 0.17). Mortality remained stable, whereas significant reductions in LOS were observed in Urology (5 to 4 days, *p* = 0.03) and traumatology (16 to 8.5 days, *p* < 0.01). During the post-intervention period, 476 stewardship recommendations were issued for 330 patients, with an acceptance rate of 76.1%. The most frequent interventions were discontinuation of antimicrobials (25.8%), transition to oral therapy (21.0%), and de-escalation (18.7%). *Conclusions*: Implementation of a CDSS in the surgical setting was associated with reduced antimicrobial consumption, a downward trend in expenditures, and high acceptance of stewardship recommendations. Mortality remained unchanged, while reductions in LOS in selected services support the safety and potential efficiency of this approach.

## 1. Introduction

Surgical site infections (SSIs) are among the most common healthcare-associated infections (HAIs). They are associated with longer post-operative hospital stays, additional surgical procedures, admission to intensive care units (ICU), and higher mortality. According to the latest Annual Epidemiological Report published by the European Center for Disease Prevention and Control (ECDC), Member States reported 19,680 SSIs from a total of 1,255,958 surgical procedures across nine types of surgery, with rates ranging from 0.6% to 9.5% depending on the type of infection [1]. However, SSIs represent only one of the many types of infections that can occur postoperatively [2]. The use of invasive devices such as intravenous (IV) lines, urinary catheters, and mechanical ventilation also increases the risk of postoperative infection in these patients [3,4]. Therefore, the management of surgical patients in the immediate postoperative period is particularly important, as is their hospital length of stay (LOS).

Optimizing antimicrobial stewardship in the surgical setting plays a key role not only in reducing the incidence of SSIs and other types of HAIs in the surgical patient, but also in limiting the emergence of antimicrobial resistance (AMR) [5]. A range of antimicrobial stewardship programs (ASPs) have been developed over recent years. These programs are designed to optimize the selection, dose, and duration of antimicrobial treatments, achieving the best clinical outcome while minimizing patient harm and ecological impact [6,7].

There is increasing evidence that ASPs are effective in improving surgical outcomes [8,9,10]. However, implementing these programs remains a major challenge. Most interventions require manual evaluation and rely on the expertise of clinical pharmacists or infectious disease specialists. Clinical Decision Support Systems (CDSSs) have become valuable tools for improving antimicrobial use and facilitating this process. CDSSs are defined as computer applications and algorithms created to guide professionals in making diagnostic and therapeutic decisions for patients [11,12]. Given the increasing healthcare demand and patient volumes, CDSS has become an area of great interest, with a wide range of potential applications. Integrated into clinical practice, CDSS can provide different services, such as access to information, statistical calculations, and specific recommendations or alerts based on patient data [6,13].

The aim of our study was to analyze the impact of a CDSS for ASP in the surgical setting, assessing antimicrobial consumption and clinical outcomes (mortality and LOS). Additionally, the study describes recommendations focused on antimicrobial management in these patients and their overall acceptance rate.

## 2. Materials and Methods

### 2.1. Study Setting

The study was conducted at Hospital Universitario Infanta Cristina, a primary-level hospital located in Parla (Madrid, Spain). The hospital has 188 beds, including surgical services such as General and Digestive Surgery, Traumatology, Obstetrics, Gynecology, Ophthalmology, Otorhinolaryngology, and Urology. The mean number of patient-days per year for these services was 13,870 for the 2018–2023 period. Approximately 75% of these patient-days corresponded to patients admitted under General and Digestive Surgery, Traumatology, and Obstetrics.

Nearly 40% of patients admitted to surgical units at our hospital receive antimicrobial therapy daily. In 2019, antimicrobial expenditures in surgical departments amounted to €37,660, representing 25.8% of the hospital’s total antimicrobial spending.

### 2.2. Antimicrobial Stewardship Program and Clinical Decision Support System

In 2020, the coordination of the ASP program at our hospital was assumed by a clinical pharmacist. The ASP team was restructured, including one pharmacist with expertise in antimicrobials, four internal medicine physicians, one microbiologist, and two intensive care physicians. The ASP protocol was also updated to incorporate new activities and indicators.

Prior to this, ASP activities were limited to the review of surgical patients who received antimicrobials classified as having “high ecological impact”: carbapenems (meropenem, imipenem/cilastatin and ertapenem), piperacillin/tazobactam, linezolid, daptomycin and tigecycline. No systematic review was conducted for other antimicrobials, and there was no methodology for selecting cases eligible for interventions. Additionally, ASP recommendations were not recorded in any dedicated application.

To address these limitations, the CDSS WASPSS^®^ (Wise Antimicrobial Stewardship Program Support System, version 1.0, developed by the University of Murcia, Murcia, Spain) was implemented in October 2020. This application integrates data from antimicrobial prescriptions, biochemistry, and microbiology results to automatically generate alerts that are reviewed by the ASP team. Based on these alerts, the team issues targeted recommendations related to antimicrobial therapy and diagnostic evaluation in surgical patients.

These alerts relate to antimicrobial treatments, including broad-spectrum agents, intravenous (IV) formulations, antimicrobials with good oral bioavailability or a narrow therapeutic index, restricted antimicrobials, and those selected by the ASP team due to ecological or safety concerns (such as carbapenems and IV fluoroquinolones). They also cover microbiological findings, including bacteremia, multidrug-resistant microorganisms, and positive blood cultures for Staphylococcus aureus, *Enterococcus* spp., *Pseudomonas aeruginosa*, *Streptococcus* spp., *Candida* spp., and *Escherichia coli*.

The system also displays demographic data and patient location within the unit, allowing users to monitor clinical information and its evolution in real time. This evolution window includes biochemistry results, alerts, ASP recommendations issued, and microbiological findings, enabling a comprehensive review of each patient’s clinical course.

### 2.3. Study Design

To evaluate the impact of the CDSS implementation on surgical patients, we conducted a quasi-experimental time series study with a pre- and post-intervention comparison. The pre-intervention period included patients admitted to surgical services in our hospital between 1 April 2017 and 30 September 2020, and the post-intervention period included those admitted between 1 October 2020 and 31 March 2024. Patients in clinical trials and those receiving antimicrobial treatments by routes other than IV or oral (inhaled, intramuscular, or topical) were excluded.

### 2.4. Study Variables

To assess antimicrobial consumption in surgical patients, monthly rates of defined daily doses (DDDs) per 1000 patient-days were compiled. DDDs were calculated as the total dose administered during hospitalization divided by the daily average maintenance dose per day for an antimicrobial drug used for its main indication in adults weighing more than 70 kg.

Changes in antimicrobial consumption before and after the implementation of the CDSS were analyzed. These changes were stratified by anatomical and therapeutic classification—ATC group (J01—antibacterials for systemic use and J02—antifungals for systemic use), considering the third level (pharmacological–therapeutic subgroup) for antibacterials. Additional stratifications included route of administration, IV antimicrobials with good oral bioavailability (doxycycline, clindamycin, cotrimoxazole, ciprofloxacin, levofloxacin, linezolid, metronidazole, fluconazole, and voriconazole), and primary service. Comparisons were also made for antimicrobials targeted by the ASP team (carbapenems and IV fluoroquinolones). Overall expenditures were calculated in €/1000 patient-days.

Clinical outcomes included in-hospital mortality (proportion of patients who died during the hospitalization) and LOS in days. These outcomes were compared between the pre- and post-intervention groups, and analyses were restricted to patients admitted for infectious reasons.

To describe ASP recommendations in surgical patients, demographic variables (sex and age) and the primary surgical service were collected. Clinical variables included type of infection (respiratory, urinary, skin and soft tissue, intra-abdominal, fever syndromes, osteoarticular, bacteremia, endovascular, and central nervous system infection), antimicrobial involved (ATC classification and route of administration), type of recommendation, clinical decision, and method of contact. The recommendation acceptance rate (%) was calculated as the number of accepted recommendations divided by the total number issued by the ASP team. Recommendations in which acceptance or rejection could not be determined (e.g., patient discharge, therapy modification for unrelated reasons, or death) were classified as “follow-up not feasible”.

Antimicrobial consumption data were extracted from the FarmaTools^®^ Unidosis software (version 3.0, Glintt Healthcare, Madrid, Spain), clinical outcomes (in-hospital mortality and LOS) from the Minimum Basic Hospital Data Set (MBDS), and data on ASP recommendations from the CDSS WASPSS^®^.

### 2.5. Statistical Analysis

Continuous variables are described using medians and interquartile ranges (IQRs), and categorical variables are expressed as absolute frequencies and percentages. Comparisons between continuous variables (antimicrobial consumption and LOS) were assessed using the Wilcoxon rank-sum test; the chi-square test or Fisher’s exact test, as appropriate, was used to compare two categorical variables (mortality). A two-sided *p*-value < 0.05 was considered statistically significant. Statistical analyses were conducted using Stata Statistical Software, Release 14 (StataCorp LP, College Station, TX, USA).

## 3. Results

### 3.1. Impact on Antimicrobial Consumption

Overall antimicrobial consumption decreased by 4.4% in the post-intervention period (from 608.7 to 581.7 DDD/1000 patient-days), although this difference was not statistically significant (*p* = 0.16). A reduction was also observed for antibacterials J01 (from 579.6 to 543.9 DDD/1000 patient-days, *p* = 0.09), whereas antifungal consumption increased after the implementation of the CDSS (from 21.5 to 28.1, *p* = 0.30). Significant relative reductions were observed for aminoglycoside antibacterials (52.0%), macrolides, lincosamides and streptogramins (40.6%), quinolone antibacterials (32.3%), and penicillins (9.2%) (Table 1). All services experienced reductions in antimicrobial consumption, except General and Digestive Surgery and Obstetrics, with no significant increases observed (Table 2). When ATC groups were analyzed by surgical service, significant reductions were observed in Traumatology (−60.3% for penicillins, *p* <0.01), Gynecology (−81.5% for aminoglycosides, *p* < 0.01), and Urology (−57.7% for quinolone antibacterials, *p* < 0.01). In obstetrics, reductions were also significant for penicillins (−17.3%, *p* < 0.01), macrolides, lincosamides and streptogramins (−57.7%, *p* < 0.01), and aminoglycosides (−62.5%, *p* < 0.01). No significant changes were observed in General and Digestive Surgery, Otorhinolaryngology, or Ophthalmology.

Overall antimicrobial expenditures decreased from 3185.4 to 2733.9 €/1000 patient-days, representing a relative reduction of 14.2% (*p* = 0.17). Although differences did not reach statistical significance (Table 3), relative reductions were observed in several surgical services: Otorhinolaryngology (−32.3%), General and Digestive Surgery (−26.9%), Traumatology (−24.2%), and Gynecology (−19.1%).

### 3.2. Impact on Clinical Outcomes

In patients admitted for infectious reasons (n= 1243), clinical outcomes were stable across periods. Overall mortality decreased slightly from 0.9% to 0.7% (*p* = 0.75), while median LOS remained at 4 days with similar IQRs (2–8 vs. 2–7, *p* = 0.29). When stratified by surgical services, no significant differences in mortality were observed, whereas significant reductions in LOS were found in Urology and Traumatology (Table 4).

### 3.3. ASP Recommendations

During the post-intervention period, a total of 476 recommendations were issued for 330 surgical patients. Demographic and clinical characteristics of these patients are presented in Table 5. The most frequent antimicrobials included in ASP recommendations were piperacillin/tazobactam (15.1%), amoxicillin/clavulanate (10.1%), metronidazole (9.5%), ciprofloxacin (8.6%), and ceftriaxone (7.8%).

Overall, 76.1% (362/476) of ASP recommendations were accepted, 6.3% (30/476) were rejected, and 17.6% (84/476) were classified as follow-up not feasible. Acceptance by primary services is shown in Table 6. Suspension, transition to oral therapy, and de-escalation accounted for nearly two-thirds of all interventions (Table 7). The most frequent mode of communication was a note in the electronic health record (65.3%; 311/476), followed by phone calls (25.4%; 121/476), face-to-face discussions (5.3%; 25/476), combined EHR note and phone call (3.0%; 14/476), and phone message (1.0%; 5/476).

## 4. Discussion

In this study, we analyzed the implementation of a CDSS to optimize antimicrobial use in the surgical setting, evaluating its impact on consumption and clinical outcomes, and describing the clinical features of ASP recommendations. Overall, antimicrobial consumption decreased, with significant reductions in specific subgroups and in targeted antibiotics such as fluoroquinolones, while carbapenem use showed a non-significant downward trend. Mortality rates remained stable during the periods, whereas significant reductions in LOS were observed in Urology and Traumatology. The use of the CDSS facilitated a high number of ASP recommendations with good acceptance, although acceptance rates varied across surgical services. To our knowledge, this is the first study assessing the outcomes of a CDSS specifically designed for ASP across different surgical services. The CDSS implemented in our setting provided structured, rule-based guidance by integrating prescribing information with biochemistry and microbiology results. These alerts directly guide providers toward optimization opportunities, such as treatment discontinuation, de-escalation, or transition to oral therapy, thereby contributing to improved antimicrobial use despite the absence of statistically significant global reductions in consumption.

A relevant finding of our study was the reduction in overall antimicrobial consumption following CDSS implementation, with a relative decrease of 4.4%. Although this global reduction did not reach statistical significance, significant decreases were observed in penicillins, macrolides/lincosamides/streptogramins, aminoglycosides, and fluoroquinolones. In our cohort, fluoroquinolone use decreased by 32.8%, consistent with previous studies showing that CDSS-supported stewardship interventions are particularly effective in reducing the use of agents associated with a high ecological footprint and frequent misuse [13,14,15,16]. Gunn et al. [17] implemented an electronic restriction and approval system coupled with a best-practice alert for fluoroquinolone prescribing in uncomplicated infections and reported a significant reduction in fluoroquinolone use (15.7% vs. 24.6% in controls, *p* < 0.01). Systematic reviews of CDSS in ASP report similar patterns, noting that these interventions commonly achieve improvements in appropriate prescribing and reductions in overused agents, although effects vary depending on CDSS design and ASP integration [13]. In addition, carbapenem use in our cohort showed a relative reduction of 19.5% although this did not reach statistical significance. This trend is in line with other experiences indicating that more targeted or restrictive interventions are often required to achieve meaningful reductions in this class [18,19].

Beyond antimicrobial classes, we found heterogeneous effects across surgical services. A significant reduction was found for quinolone antibacterials in Urology (−57.7%), with a high clinical relevance as these drugs are linked with serious side effects and contribute to bacterial resistance in urinary tract infections [20]. Significant decreases were also observed in Traumatology, Gynecology, and Obstetrics for penicillin derivatives, macrolides, and aminoglycosides, respectively. These discrepancies across surgical services reflect the underlying diversity in types of infections and empirical regimens. For instance, the stable consumption in General and Digestive Surgery may be explained by the frequency of beta-lactam/beta-lactamase inhibitor combinations for intra-abdominal infections, whereas the marked reductions in Urology and Obstetrics are consistent with ASP efforts targeting fluoroquinolones and aminoglycosides, due to safety reasons.

Several studies have highlighted the variability and lack of adherence to antimicrobial prescribing guidelines among surgeons, which may partly explain this finding. An international survey revealed significant differences in surgical antimicrobial prophylaxis practices across countries and specialties [21]. Similarly, local analyses have documented poor compliance with established guidelines, even in high-volume general surgery departments [22,23]. These observations underscore the challenges of translating evidence-based recommendations into routine surgical practice and reinforce the importance of targeted, specialty-specific stewardship interventions to ensure standardized implementation. In this regard, our results emphasize the need to tailor stewardship strategies to the specific context of each surgical department.

Regarding expenditure, although the overall reduction of 14.2% did not reach statistical significance, the observed downward trend is consistent with previous reports of CDSS-based ASP. SE Bond et al. [24] demonstrated that implementation of a collaborative multisite ASP supported by a centrally deployed CDSS significantly reduced antimicrobial use and decreased antimicrobial costs by AUD 64,551 per month (17%; *p* < 0.01). Nault et al. [25] also reported the sustained impact of a computer-assisted antimicrobial stewardship intervention in Canada, with antimicrobial costs reduced by 33.6% using variable pricing and by 28.3% using fixed pricing, corresponding to direct annual savings of approximately CAD 350,000. Taken together, these experiences are consistent with our findings, suggesting that although the magnitude of savings may vary across settings, CDSS can contribute to more efficient antimicrobial use and generate substantial economic benefits.

Hospital mortality remained very low and stable after CDSS implementation, supporting the safety of this intervention. This trend is consistent with the literature, where reported reductions are modest and often not statistically significant. Carracedo-Martínez et al. [26] highlighted the difficulty of demonstrating a positive effect on mortality, as improvements in antimicrobial prescribing are frequently diluted by other clinical factors, which can influence patient outcomes. Similarly, Rittmann et al. [12] described only inconsistent effects on mortality across studies.

In contrast, significant reductions in LOS were observed in Urology and Traumatology, suggesting that the clinical impact of CDSS may not be uniform but rather dependent on patient characteristics and the type of infectious processes involved. These services are particularly exposed to the post-operative antimicrobials, which reinforces the impact of the observed benefit. In the literature, reductions in LOS have been reported more frequently in medical infections, while evidence in surgical settings remains scarce. Yuan et al. [27] likewise found no differences in median LOS among surgical patients, whereas significant reductions were noted in medical infections such as respiratory, urinary, and skin and soft tissue infections. Our findings therefore support the efficient use of the CDSS to improve clinical outcomes in selected surgical services.

Although most differences in results did not reach statistical significance, the consistent downward trends across antimicrobial classes (including those with ecological or toxicity concerns) and the reductions in LOS suggest a clinically meaningful impact. These findings reflect the complexity of these interventions in real practice, where statistical significance may be difficult to accomplish due to population heterogeneity and epidemiological factors, yet the directionality and safety reasons support the value of the intervention.

The implementation of the CDSS in surgical patients resulted in 476 ASP recommendations. The most frequently involved services were General and Digestive Surgery (41.8%) and Urology (29.4%). This distribution differs from that reported by Batlle et al. [28], where almost all recommendations were focused on General and Digestive Surgery (90.7%), and a minority in Urology (8.1%). Similar to their findings, the most frequently intervened antibiotics in our study were piperacillin/tazobactam (15.1%), amoxicillin/clavulanate (10.1%), and metronidazole (9.5%), supporting their role in treating perioperative and postoperative infections.

ASP recommendations were mostly associated with IV treatments (92.7%). This finding highlights the importance of ASP strategies centered on IV antimicrobials, since transition to oral therapy can reduce cost, LOS, and complications related to IV administration, as endorsed by the Infectious Diseases Society of America (IDSA) and Society for Healthcare Epidemiology of America (SHEA) [29].

Acceptance of ASP recommendations by primary providers was 76.1%, with only 6.3% being rejected. This proportion, although lower than that reported by Batlle et al. [28] (91.4%), should be interpreted in the context of their study, which included only antimicrobial courses exceeding 7 days. Our results are in line with the acceptance rates commonly described in the literature, ranging from 70 to 90% [30,31,32,33], and are particularly relevant as they focus solely on surgical patients, who are variably represented in other CDSS evaluations. Acceptance varied across surgical services, with Gynecology (95.1%) and General and Digestive Surgery (82.4%) showing the highest rates, emphasizing these areas for the implementation of ASP activities supported by CDSS.

The most frequent recommendations were suspension (25.8%), transition to oral therapy (21.0%), and de-escalation (18.7%). These proportions are slightly different from those reported by Neuner et al. [34] in medical patients, where discontinuation (37%) and de-escalation (28%) prevailed. There is scarce published evidence specifically describing the types of ASP recommendations in surgical patients, despite their increased risk of antimicrobial overuse or inappropriate use in the post-operative setting [5].

Our study has several limitations. First, the implementation of the CDSS in the hospital coincided with the coronavirus disease pandemic, which could have increased the number of nosocomial infections in surgical patients. However, mortality and LOS for surgical patients admitted for infectious reasons did not show significant changes that could be attributed to this issue. Second, its quasi-experimental and single-center design could affect the applicability and reliability of the findings, considering that the use of these tools in this setting is limited and published evidence remains sparse. Third, the incidence of multidrug-resistant organisms could not be stratified by surgical service because microbiology data were only available at the hospital level in the pre-implementation period. Therefore, the potential influence of MDR on antimicrobial use patterns could not be assessed for this population. Finally, the appropriateness of antimicrobial prescriptions in our patients could not be evaluated, as the only measure used was DDDs, which reflects consumption and use patterns but not adequacy. Nonetheless, consumption tended to decrease in almost all services, suggesting a positive effect of the ASP activities.

## 5. Conclusions

In conclusion, the implementation of a CDSS for ASP in the surgical setting was associated with reductions in antimicrobial consumption, including significant decreases in several drug classes and in Traumatology and Urology services. In addition, a downward trend in antimicrobial expenditures was observed, suggesting a potential economic benefit. Mortality remained low and LOS stable overall, with reductions observed in specific surgical specialties, supporting the safety and potential efficiency of this approach. ASP recommendations achieved high acceptance rates and provided actionable information to optimize management in surgical patients. Further research is needed to confirm these findings and to assess their applicability across different hospitals and surgical settings.

## Figures and Tables

**Table 1:** Differences in antimicrobial consumption by ATC group and specific classes (DDDs/1000 patient-days).

	Pre-Intervention Period	Post-Intervention Period	*p* Value
Overall antimicrobials	608.7	581.7	0.16
Tetracyclines (J01A)	5.2	12.0	**<0.01**
Beta-lactam antibacterials, penicillins (J01C)	221.6	201.1	**0.01**
Other beta-lactam antibacterials (J01D)	156.2	160.8	0.24
Sulfonamides and trimethoprim (J01E)	0.6	0.0	0.18
Macrolides, lincosamides, and streptogramins (J01F)	40.1	20.5	**<0.01**
Aminoglycoside antibacterials (J01G)	27.1	13.0	**<0.01**
Quinolone antibacterials (J01M)	51.8	35.1	**<0.01**
Other antibacterials (J01X)	58.8	87.9	**<0.01**
IV antimicrobials	466.3	451.6	0.49
Oral antimicrobials	130.6	115.7	0.14
IV with good oral bioavailability	66.1	71.8	0.89
Carbapenems	35.4	28.5	0.18
IV fluoroquinolones	19.8	13.3	**<0.01**

IV: intravenous. Values in bold indicate statistically significant differences (*p* < 0.05).

**Table 2:** Differences in antimicrobial consumption by primary service (DDDs/1000 patient-days) ^1^.

	Pre-Intervention Period	Post-Intervention Period	*p* Value
General and Digestive Surgery	708.0	793.8	0.08
Traumatology	646.1	508.7	**<0.01**
Obstetrics	244.7	254.6	0.61
Gynecology	728.1	544.7	0.33
Otorhinolaryngology	630.2	576.8	0.49
Urology	712.4	610.9	**0.02**

^1^ Ophthalmology was not included as it showed a median of 0.0 in both periods. Values in bold indicate statistically significant differences (*p* < 0.05).

**Table 3:** Differences in antimicrobial expenditures (€/1000 patient-days) ^1^.

	Pre-Intervention Period	Post-Intervention Period	*p* Value
General and Digestive Surgery	7210.3	5273.5	0.10
Traumatology	1675.2	1269.8	0.16
Obstetrics	462.8	492.2	0.75
Gynecology	1874.4	1515.9	0.24
Otorhinolaryngology	2619.0	1775.1	0.13
Urology	2143.7	2573.6	0.66

^1^ Ophthalmology was not included as it showed a median of 0.0 in both periods. Values in bold indicate statistically significant differences (*p* < 0.05).

**Table 4:** Mortality and LOS by primary service.

Primary Service ^1^	Variable	Pre-Intervention Period	Post-Intervention Period	*p* Value ^2^
Urology (n = 342)	Mortality, %	0.6	0.5	0.91
	LOS, median (IQR)	5 (3–8)	4 (3–7)	**0.03**
Otorhinolaryngology (n = 235)	Mortality, %	0.0	0.0	N/A
	LOS, median (IQR)	1 (1–3)	1 (1–3)	0.46
Gynecology (n = 230)	Mortality, %	0.0	0.0	N/A
	LOS, median (IQR)	3 (2–5)	3 (2–5)	0.37
General and Digestive Surgery (n = 199)	Mortality, %	1.8	1.1	0.70
	LOS, median (IQR)	7 (3–15)	6 (3–10)	0.70
Traumatology (n = 153)	Mortality, %	3.9	2.6	0.66
	LOS, median (IQR)	16 (9–26)	8.5 (4.5–16)	**<0.01**
Obstetrics (n = 84)	Mortality, %	0.0	0.0	N/A
	LOS, median (IQR)	3 (2–4)	2 (2–3)	0.20

^1^ Ophthalmology was not included as it showed a median of 0.0 in both periods. ^2^ N/A: Not applicable. Values in bold indicate statistically significant differences (*p* < 0.05).

**Table 5:** Demographic and clinical characteristics of patients with ASP recommendations.

Characteristic	n (%)
Demographic data	
Age, median (IQR)	67 (51–76)
Sex (men)	190 (57.6)
Primary service	
General and Digestive Surgery	199 (41.8)
Urology	140 (29.4)
Traumatology	71 (14.9)
Gynecology	41 (8.6)
Otorhinolaryngology	11 (2.3)
Obstetrics	8 (1.7)
Ophthalmology	6 (1.3)
Type of infection	
IAI	157 (32.9)
UTI	145 (30.4)
SSTI	104 (21.9)
Osteoarticular	41 (8.6)
Respiratory	15 (3.2)
Bacteremia	6 (1.3)
Endovascular	5 (1.1)
Fever syndromes	2 (0.4)
CNS infection	1 (0.2)
Antimicrobial drug	
Other antibacterials (J01X)	128 (26.9)
Beta-lactam antibacterials, penicillins (J01C)	121 (25.4)
Other beta-lactam antibacterials (J01D)	106 (22.3)
Quinolone antibacterials (J01M)	54 (11.3)
Aminoglycoside antibacterials (J01G)	28 (5.9)
Antifungals for systemic use (J02A)	21 (4.4)
Tetracyclines (J01A)	11 (2.3)
Macrolides, lincosamides, and streptogramins (J01F)	7 (1.5)
Route of administration	
Intravenous	441 (92.7)
Oral	37 (7.3)

CNS: central nervous system; IAI: intra-abdominal infection; SSTI: skin and soft tissue infection; UTI: urinary tract infection.

**Table 6:** Acceptance of ASP recommendations by the primary service.

Primary Service	Accepted (n)	Rejected (n)	Follow-Up Not Feasible (n)	Acceptance Rate (%)
General and Digestive Surgery	164	10	25	82.4
Urology	96	11	33	68.6
Traumatology	52	7	12	73.2
Gynecology	39	0	2	95.1
Otorhinolaryngology	6	1	4	54.5
Obstetrics	2	0	6	25.0
Ophthalmology	3	1	2	50.0

**Table 7:** Type of ASP recommendation.

Type of Recommendation	n (%)
Suspension	123 (25.8)
Transition to oral therapy	100 (21.0)
De-escalation	89 (18.7)
Dose adjustment	47 (9.9)
Substitution to another antimicrobial	37 (7.8)
Drug level monitoring	34 (7.1)
Interval adjustment	25 (5.3)
Duration adjustment	11 (2.3)
Adjustment of concomitant medication	7 (1.5)
Start of new antimicrobial	3 (0.6)

## Data Availability

The data underlying this article are not publicly available due to patient privacy and institutional restrictions. Aggregated anonymized data may be made available from the corresponding author upon reasonable request and with permission of Hospital Universitario Infanta Cristina.

## Abbreviations

The following abbreviations are used in this manuscript:

AMR	Antimicrobial Resistance
ASP	Antimicrobial Stewardship Program
ATC	Anatomical and Therapeutic Classification
AUD	Australian dollars
CAD	Canadian dollars
CDSS	Clinical Decision Support System
ECDC	European Center for Disease Prevention and Control
DDD	Daily Defined Dose
HAI	Healthcare-associated infections
ICU	Intensive Care Unit
IDSA	Infectious Diseases Society of America
IQR	Interquartile Range
IV	Intravenous
LOS	Length of stay
MBDS	Minimum Basic Hospital Data Set
SHEA	Society for Healthcare Epidemiology of America
SSI	Surgical site infections
WASPSS	Wise Antimicrobial Stewardship Program Support System
